# Opsin evolution and expression in Arthropod compound Eyes and Ocelli: Insights from the cricket *Gryllus bimaculatus*

**DOI:** 10.1186/1471-2148-12-163

**Published:** 2012-08-30

**Authors:** Miriam J Henze, Kara Dannenhauer, Martin Kohler, Thomas Labhart, Matthias Gesemann

**Affiliations:** 1 Department of Biology, Lund University, Sölvegatan 35, 22362, Lund, Sweden; 2 Institute of Molecular Life Sciences, University of Zurich, Winterthurerstrasse 190, 8057, Zürich, Switzerland

**Keywords:** Opsin, Visual pigment, Spectral sensitivity, Compound eye, Dorsal rim area, Ventral band, Ocellus, Insect, Orthoptera, *Gryllus bimaculatus*

## Abstract

**Background:**

Opsins are key proteins in animal photoreception. Together with a light-sensitive group, the chromophore, they form visual pigments which initiate the visual transduction cascade when photoactivated. The spectral absorption properties of visual pigments are mainly determined by their opsins, and thus opsins are crucial for understanding the adaptations of animal eyes. Studies on the phylogeny and expression pattern of opsins have received considerable attention, but our knowledge about insect visual opsins is still limited. Up to now, researchers have focused on holometabolous insects, while general conclusions require sampling from a broader range of taxa. We have therefore investigated visual opsins in the ocelli and compound eyes of the two-spotted cricket *Gryllus bimaculatus*, a hemimetabolous insect.

**Results:**

Phylogenetic analyses place all identified cricket sequences within the three main visual opsin clades of insects. We assign three of these opsins to visual pigments found in the compound eyes with peak absorbances in the green (515 nm), blue (445 nm) and UV (332 nm) spectral range. Their expression pattern divides the retina into distinct regions: (1) the polarization-sensitive dorsal rim area with blue- and UV-opsin, (2) a newly-discovered ventral band of ommatidia with blue- and green-opsin and (3) the remainder of the compound eye with UV- and green-opsin. In addition, we provide evidence for two ocellar photopigments with peak absorbances in the green (511 nm) and UV (350 nm) spectral range, and with opsins that differ from those expressed in the compound eyes.

**Conclusions:**

Our data show that cricket eyes are spectrally more specialized than has previously been assumed, suggesting that similar adaptations in other insect species might have been overlooked. The arrangement of spectral receptor types within some ommatidia of the cricket compound eyes differs from the generally accepted pattern found in holometabolous insect taxa and awaits a functional explanation. From the opsin phylogeny, we conclude that gene duplications, which permitted differential opsin expression in insect ocelli and compound eyes, occurred independently in several insect lineages and are recent compared to the origin of the eyes themselves.

## Background

Visual opsins are key proteins in animal photoreception. They belong to the superfamily of G-protein coupled transmembrane receptors and form visual pigments together with a light-sensitive prosthetic group, the chromophore. Visual pigments mediate the first step in the visual signaling pathway, the conversion of light into an electrical response. As the spectral absorption properties of a visual pigment are mainly determined by the amino acid sequence of its opsins, the fate of an opsin is related to both the function and the history of a photoreceptive structure. Studying the phylogeny and expression pattern of opsins is thus crucial for understanding the evolution of animal eyes.

Even though questions concerning the evolutionary history of opsins have lately received considerable attention (for a recent review see [1]), we still have very limited knowledge of insect visual opsins. Earlier studies have shown that they fall into three major clades (e.g. [2]): (1) UV-sensitive short-wavelength (SW) opsins, (2) blue-sensitive middle-wavelength (MW) opsins and (3) so-called long-wavelength (LW) opsins, for which the spectral sensitivities are more variable, ranging from blue-violet (Rh2 in *Drosophila*[3]) through green to red (e.g. in some butterflies [4]). Most insect species investigated so far possess at least one opsin of each type. However, previous research has focused on holometabolous insect orders (Hymenoptera e.g. [5], Diptera e.g. [6], Lepidoptera e.g. [7] and Coleopteran e.g. [8]). Some crustacean (Branchiopoda e.g. [9,10], Ostracoda e.g. [11], Malacostraca e.g. [12,13]) and three chelicerate species (two spiders [14] and a horseshoe crab [15-17]) have also been studied. But comprehensive data on earlier-branching insect lineages are missing, which makes generalized conclusions questionable.

**Figure 1:**
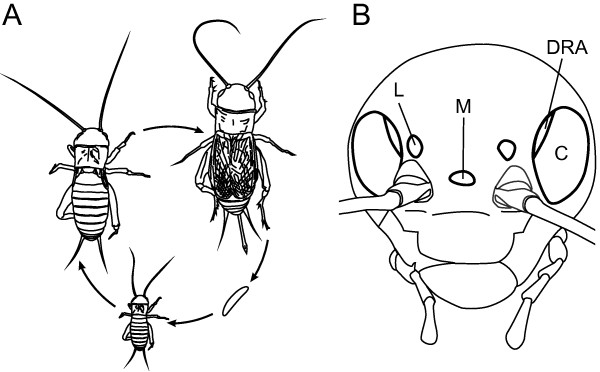
**Life cycle and visual organs of the two-spotted cricket *G. bimaculatus. *** (**A**) Crickets are hemimetabolous insects. They hatch from the egg as a larva that is a miniature version of the adult except for a lack of wings and functional reproductive organs. From one instar to the next, i.e. during molts, *G. bimaculatus* larvae grow larger, reaching adulthood after 8 to 11 molts with a weight gain of up to 1000 times [114]. (**B**) In both larvae and adults, the lateral ocelli (L) are positioned dorsal to the antennal bases, and the median ocellus (M) is located on the forehead. Each compound eye (**C**) features a specialized dorsal rim area (DRA) and a ventral band (not marked, as its exact position is not known). Eye size increases from one larval stage to the next by adding new ommatidia along a budding zone at the anterior margin of the eye [42-44].

To close this gap, we have investigated visual opsins in the two-spotted cricket, *Gryllus bimaculatus* (Orthoptera), an important model organism in neurobiological, physiological, developmental and regeneration research [18]. Crickets are hemimetabolous insects, i.e. insects without a pupal stage and without dramatic changes in body plan from the larva to the adult (Figure 1A). Their developmental program fundamentally differs from the one of the previously studied holometabolous insects, which undergo a complete metamorphosis from larva to imago during pupal stage. The ancestors of modern crickets diverged about 350 million years ago (mya) from the branch that gave rise to holometabolous insects [19,20]. Our results thus provide a link between the studies on Holometabola and those on non-hexapod arthropods such as crustaceans and chelicerates.

Like many other adult insects, crickets possess two kinds of visual organs (Figure 1B): a pair of lateral compound eyes innervated from the optic lobes and three dorsal ocelli innervated from the posterior part of the central brain [21]. Both eye types were probably present in the first euarthropods already [22-24], which indicates that their evolutionary divergence dates back at least to the early Cambrian, more than 500 mya [25]. While ocelli are cup-shaped single-lens eyes that were inherited from arthropod predecessors [22,24,26], compound eyes are a novel acquisition for euarthropods [22-24,27]. They consist of replicated subunits, the ommatidia, which are basically identical but can be modified in some respects to create retinal heterogeneity and regionalization [27-29]. In holometabolous insects, such as flies, the development of compound eyes and ocelli is delayed until adulthood [30]. Larvae can have a number of simple eyes, termed stemmata (or Bolwig organs in *Drosophila*), which share common ancestry with the compound eyes [31,32]. In contrast, hemimetabolous insects, such as crickets, hatch from the egg already equipped with well-developed compound eyes and ocelli. Each ocellus has a single lens consisting of transparent cuticle. A clear zone is located between it and the underlying photoreceptor layer, which comprises a large number of closely packed retinula cells in an irregular arrangement [33,34]. In the two-spotted cricket, the lateral ocelli are positioned just dorsal to the antennal bases, whereas the median ocellus is located on the forehead (Figure 1B). As in most insect species, the function of the ocelli is not well-studied in crickets. The lens optics and retinal structure suggest that the ocelli of *G. bimaculatus* are heavily under-focused with poor image quality (T Labhart, unpublished observations). A number of investigations in different cricket species proposed that the ocelli play a role in compound eye adaptation [35], entrainment of circadian rhythms ([36]; but see [37,38]) and phototaxis [39]. In other insects, ocelli provide compass cues based on polarized skylight (reviewed by [40]) or are horizon detectors that serve the control of head orientation and stabilize flight posture (reviewed by [41]).

The compound eyes are the main visual organs of the two-spotted cricket. They grow from one larval stage to the next by the addition of new ommatidia along a budding zone at the anterior margin of the eye [42-44]. In the adult, a compound eye comprises about 4600 ommatidia [43]. Each ommatidium consists of a corneal lens, two primary and several secondary pigment cells, four crystalline cone cells and eight receptor cells. The rhabdomeres of the photoreceptors fuse to form a closed rhabdom which is directly connected to the crystalline cone [45]. Despite this uniform design, the fine structure of the ommatidia varies. A specialized region at the dorso-frontal margin of the compound eye, the so-called dorsal rim area (DRA, Figure 1B), can easily be detected, even in the live cricket, because of its pale appearance. The ommatidia that constitute the DRA lack well-defined facets and screening pigment, and the pigment cells are vestigial [45-47]. As in many other insects, the DRA in crickets is a non-imaging eye region [48-50]. Anatomical, electrophysiological and behavioral experiments have shown that the cricket DRA samples polarized skylight information over a large part of the celestial hemisphere for orientation [45,51-56]. Apart from polarization vision, insect compound eyes generally fulfill a number of different functions including intensity discrimination, motion vision, distance and form perception and, at least in some species, color vision [57].

Here, we have cloned four visual opsin genes of the two-spotted cricket. We analyze their phylogenetic relationship in a broad context by including data on other insect and non-hexapod arthropod species. Furthermore, we describe the spatial expression pattern of the cricket opsin paralogs in the compound eyes and ocelli of cricket larvae and adults based on *in situ* hybridizations. We also determine the spectral sensitivities of the respective visual pigments by referring to previous intracellular recordings of compound eye photoreceptors [54] and our own electroretinogram (ERG) recordings of the ocelli. In this paper, we thus present the first comprehensive study on visual opsins in a hemimetabolous insect.

## Methods

### Animals

A population of two-spotted crickets (*Gryllus bimaculatus,* De Geer) was founded in 2004 by specimens collected in Tunisia in May and September at field locations close to Sidi Bou Saïd (N 36° 52’, E 10° 20’), Tebersouk (N 36° 27’, E 9° 15’), Kasserine (N 35° 10’, E 8° 49’) and Café Bir Soltane (N 33° 17’, E 9° 44’). The animals were subsequently maintained and bred in the laboratory under a 14/10 hours light–dark cycle (L20W/10S daylight lamps; Osram) at 26°C and 60% relative humidity. All experiments reported here were carried out between February 2007 and November 2008 on descendants of the wild-caught founder individuals.

### Cloning and sequencing

Cricket larvae and adults of both sexes were rapidly frozen in liquid nitrogen and total RNA was extracted from the head or, in case of the UV opsin, from the pigmented part of the compound eye using the RNeasy kit (Qiagen). The RNA was reverse transcribed by the SuperScript First-Strand Synthesis System for RT-PCR (Invitrogen). *Gb* (*Gryllus bimaculatus*) opsin sequences were amplified by PCR (polymerase chain reaction) using sets of degenerate primers designed on conserved, lowly degenerated amino acid codons of other insect opsins. PCR products of the predicted length were ligated into the pCRII vector (Invitrogen) and sequenced. Based on these results, gene-specific primers were designed to carry out a 5’ and 3’ RACE (rapid amplification of cDNA ends) according to the manufacturer’s instructions (for details see Invitrogen No. 18374-058 and 18373-019). RACE products were sequenced and overlapping opsin sequences assembled in the SeqMan module of Lasergene (DNAStar). At least three independently amplified cDNA fragments were sequenced for each nucleotide position. Additionally, full-length opsin sequences were amplified by gene-specific primers and verified. All primers used for amplification are listed in Additional file 1. The cricket opsin sequences reported in this paper have been deposited in the GenBank database [58]: *Gb* GreenA/OpsinLWa [GenBank:HM363620], *Gb* GreenB/OpsinLWb [GenBank:HM363621], *Gb* Blue/OpsinMW [GenBank:HM363622], *Gb* UV/OpsinSW [GenBank:HM363623].

### Phylogenetic analysis

*Gb* opsins and other arthropod opsin sequences downloaded from GenBank [58] or identified in a genome assembly [59] were analyzed on the Phylogeny.fr platform [60]. We included two chelicerates (a spider and a horseshoe crab), a branchiopod crustacean, representatives of several insect orders (Hemiptera, Coleoptera, Hymenoptera, Diptera and Lepidoptera) and all orthopteroid insect species (Mantodea and Orthoptera) for which full-length sequence information was available (for references see Additional file 2; FASTA-formatted sequences are provided in Additional file 3). Three different, uncontroversial outgroups were chosen in alternative analyses. Translated amino acid sequences were aligned using MUSCLE v3.7 [61] configured for highest accuracy (MUSCLE with default settings). After alignment, ambiguous regions (i.e. regions containing gaps and/or being poorly aligned regions) were removed with Gblocks v0.91b [62] leaving 224, 240 or 233 out of 483 positions depending on whether honeybee (*Apis mellifera*) pteropsin, zebrafish (*Danio rerio*) melanopsin or Hawaiian bobtail squid (*Euprymna scolopes*) eye opsin was specified as outgroup. Phylogenetic trees were reconstructed using the maximum likelihood method implemented in the PhyML program v3.0 [63]. The WAG amino acid substitution model [64] was selected assuming an estimated proportion of invariant sites and 4 gamma-distributed rate categories to account for rate heterogeneity across sites. The gamma shape parameter was estimated directly from the data. Branch reliability was assessed by the approximate likelihood-ratio test (aLRT, SH-like) [65]. Graphical representations of the phylogenetic trees were obtained using TreeDyn v198.3 [66] and edited in Adobe Illustrator (Adobe Systems Incorporated).

### *In situ* hybridization

The following steps were modified from an *in situ* hybridization protocol originally developed for zebrafish [67]. Sense and antisense digoxigenin (DIG) labeled RNA probes were transcribed from linearized plasmids containing a cDNA fragment of the respective *Gb* opsin gene using the DIG RNA labeling kit (Roche Diagnostics). The probes comprised the following positions of the coding sequence: 109 to 773 and 109 to 890 for *uv*, 204 to 782 and 204 to 885 for *blue*, 419 to 872 for *greenA* and 145 to 869 for *greenB*. Cricket heads were fixed in 4% paraformaldehyde at room temperature for 45 minutes and subsequently embedded in freezing medium (Tissue-Tek O.C.T. Compound, Sakura Finetek Europe B.V.) and frozen in liquid nitrogen. 15 to 20 μm thick sections were cut on a cryostat (Microm HM 550, Thermo Fisher Scientific Inc.), mounted on silane coated slides and dried at 65°C. The tissue was postfixed in 4% paraformaldehyde for 30 minutes. Following acetylation and washing steps, the slides were equilibrated in hybridization buffer (50% formamide, 5% Denhardt's solution, 750 mmol/l NaCl, 75 mmol/l trisodium citrate dihydrate, 0.5 mg/ml herring sperm DNA, 0.25 mg/ml torula yeast RNA) for 3 hours and then incubated overnight at 58°C with approximately 3 ng/μl labeled probe in hybridization buffer. After further washing steps, the tissue was treated with blocking solution (3% skim milk powder) for three hours. An alkaline phosphatase-coupled anti-Dig Fab-fragment (Roche Diagnostics) applied in blocking solution was allowed to bind to the labeled probe for two hours. Finally, a colorimetric reaction with the two substrates nitro blue tetrazolium (NBT) and 5-bromo-4-chloro-3-indolyl phosphate (BCIP) in alkaline phosphatase solution containing 50 mmol/l MgCl_2_ and 1 mmol/l levamisole was used to detect bound Dig-labeled Fab fragments. Images of the processed sections were collected by a Color view IIIu camera mounted on a BX61 microscope (both: Olympus), and Adobe Photoshop (Adobe Systems Incorporated) was used to adjust brightness and contrast. In total, the eyes of more than 15 larvae (23 to 40 days after hatching; instars 4 to 7 approximately) and 16 adults (9 females and 7 males) were examined.

### Electroretinogram (ERG)

Adult crickets were mounted in a tight plastic tube on a holder in such a way that only the head was exposed. Both the head and the antennae were firmly glued to the tube with wax and the compound eyes were covered with opaque black emulsion paint (Herbol GmbH). The animal was transferred to a Faraday cage and an electrolytically sharpened tungsten electrode was inserted into the margin of the median or the left ocellus, while the reference electrode was positioned in the dorso-caudal part of the head capsule. ERG signals were recorded with a P15 amplifier (bandwidth 0.3-100 Hz, Grass Technologies) and monitored on the screen of a storage oscilloscope.

For each ocellus, measurements were performed under two conditions: (1) dark-adaptation and (2) light-adaptation with bright long-wavelength (LW) illumination (λ > 545 nm, edge filter, Schott AG). The ocellus was stimulated by 100 ms flashes of quasi-monochromatic light. This was achieved by passing light from a 450 W xenon arc lamp through one of thirteen narrowband interference filters ranging from 318 to 664 nm (Balzers AG). The light beam was focused into a flexible UV-transmitting light guide whose far end was positioned in the Faraday cage, where it provided a 28° stimulus centered on the ocellus under investigation. The intensity of the stimulus was adjusted by neutral density filters (Balzers AG) such that the amplitude of the ERG response was the same at all wavelengths. Spectral sensitivities were provided by the reciprocal values of the stimulus intensities and were normalized to the maximal spectral sensitivity determined for each ocellus under the respective adaptation condition.

### Statistics and models

To investigate whether the spectral sensitivities of the median and the left ocellus could be pooled for all individuals or whether there were significant differences, we used a mixed model approach to the analysis of repeated measures (MIXED procedure in SAS 9.1.3, SAS Institute Inc.). The repeated variables *ocellus* and *wavelength* were treated as fixed effects. Based on restricted maximum likelihood information criteria [68], we chose unstructured and first-order auto-regressive covariance structures for *ocellus* and *wavelength*, respectively [69]. The denominator degrees of freedom for the tests of the fixed effects were computed by the Kenward-Roger method [70] and the covariance parameter *cricket*, a random effect, was analyzed by likelihood-ratio statistics [71].

The wavelength of peak sensitivity (λ_max_) was obtained from the ERG measurements by fitting templates for the α-band of an 11-cis retinal visual pigment [72] to the data [73,74].

## Results

### Cricket opsin paralogs cluster in the three visual opsin clades of insects

**Figure 2:**
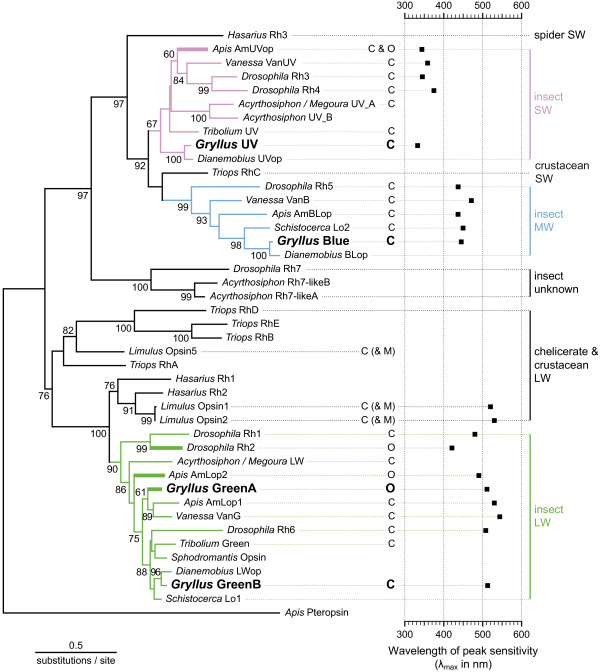
**Evolutionary origin of cricket opsins.** The phylogenetic tree is based on aligned, full-length amino acid sequences and was reconstructed using the Maximum likelihood method with honeybee pteropsin as an outgroup. No significant changes in tree topology were observed if other outgroups were used (Additional file 4). Numbers at the nodes indicate aLRT (approximate likelihood-ratio test) values for all nodes supported by more than 50%. *G. bimaculatus* sequences are highlighted in bold. The different insect opsin lineages are colored in violet (SW = short-wavelength clade), blue (MW = middle-wavelength clade) and green (LW = long-wavelength clade). If known, the wavelength of peak sensitivity (λ_max_) of the respective visual pigment is given. The location of opsin expression is indicated by C for the compound eyes, and O and bold branches for the ocelli. M in brackets stands for the median eyes of *Limulus*, for which ambiguous results exist. For species names and references on sequences, expression data and λ_max_ values see Additional file 2 and Additional file 3.

To add to the understanding of insect opsin expression and evolution, we have cloned visual opsins in a hemimetabolous insect, the two-spotted cricket *Gryllus bimaculatus* (*Gb*). Four distinct opsin encoding mRNAs were identified. The deduced proteins vary in length from 377 to 379 amino acids. In order to clarify their evolutionary origin, we have reconstructed a molecular phylogenetic tree of arthropod visual opsins based on the Maximum likelihood algorithm (Figure 2; see also Additional File 4). The cricket sequences cluster in the main visual opsin clades of insects: one in the short-wavelength (SW), one in the middle-wavelength (MW) and two in the long-wavelength (LW) branch of the phylogenetic tree. Alternative phylogenetic analyses applying the Neighbor-joining and the Bayesian method did not reveal significant differences in tree topology (data not shown).

Considering the spectral sensitivities of the compound eyes [53,54] and ocelli of crickets (see below), as well as the spatial pattern of opsin expression (see below), the *Gb* SW, MW and LW sequences can most likely be assigned to UV-, blue- and green-sensitive visual pigments and were thus termed UV, Blue, GreenA and GreenB. This classification is also supported by a known spectral tuning site. It has been shown that lysine at the position homologous to glycine 90 (G90) in bovine rhodopsin is responsible for the UV absorption properties of invertebrate SW pigments [75]. Correspondingly, lysine is found at G90 in *Gb* UV, whereas glutamate is present at G90 in *Gb* Blue, similar to most other blue opsins identified in insects so far.

### Cricket compound eyes

#### The retina is spectrally divided into three distinct regions

Previous intracellular recordings revealed three spectral classes of photoreceptors with maximal sensitivities at 332 nm (UV), 445 nm (blue) and 515 nm (green) in the compound eyes of *G. bimaculatus*[54]. Blue receptors were only found in the DRA, UV receptors only in the dorsal region of the pigmented part of the eye and green receptors everywhere outside the DRA. We have investigated the spatial pattern of opsin mRNA expression by *in situ* hybridization, and our results suggest that the photoreceptor distribution is more complex than previously assumed.

**Figure 3:**
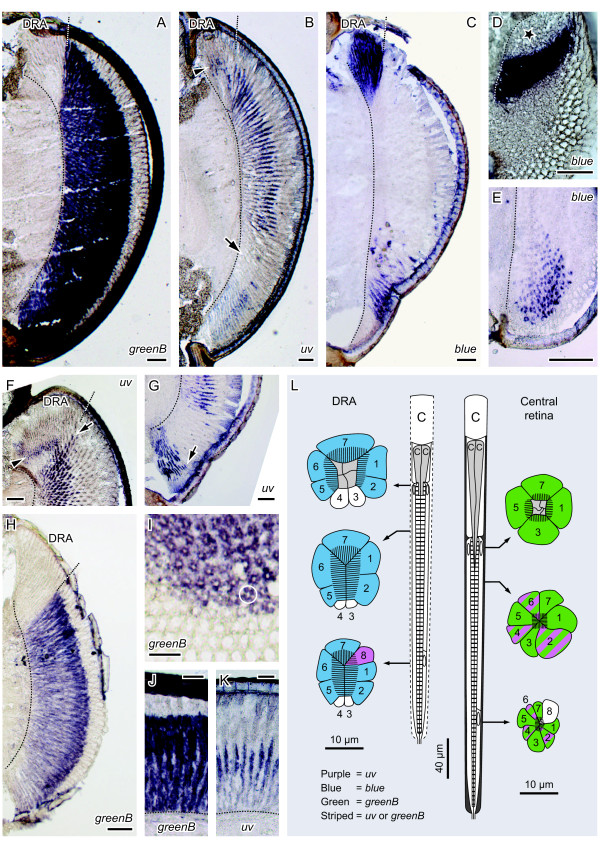
**Opsin transcripts in the cricket compound eye detected by antisense riboprobes.** (**AC**) Dorso-ventral sections through the adult retina taken at similar depths: *greenB* is transcribed in all eye regions except for the DRA; *uv* is found in the DRA (arrowhead) and in the remainder of the eye excluding a horizontal band in the ventral half (arrow); *blue* transcription is confined to the DRA and a ventral band. (**DE**) Oblique cross-sections through the dorsal-(**D**) and ventral (**E**) retina. All distal receptors in the DRA and some in the ventral retina transcribe *blue*. The asterisk denotes the growth zone of the larval eye. (**F**) Oblique cross-section-(**G**) quasi-longitudinal section through the ventral retina. *uv* is transcribed in small, proximal receptors in the DRA (arrowhead). Next to the DRA and ventral to the ventral band, *uv* can be found at more distal levels (arrows) than in the central retina. (**H**) Longitudinal section showing a typical *greenB* staining, which is stronger distally than proximally. (**I**) Distal cross-section and (**JK**) longitudinal sections through the central retina. All four receptors around the crystalline cone (as outlined by a circle) transcribe *greenB*. At the level of weaker *greenB* staining (**J**), the *uv* staining begins (**K**). Up corresponds to dorsal in (**AC**, **EH**), frontal in (**D**), medial in (**I**) and distal in (**JK**). Sections (**D**, **HI**), (**AB**, **JK**) and (**C**, **EG**) from larvae, adult females and adult males, respectively. Broken lines in histological sections denote the basement membrane and scale bars indicate 100 μm. (**L**) Schematic longitudinal sections through ommatidia in the DRA and central retina with cross-sections at indicated levels-Illustration modified after [45,46,76]. C cornea, CC crystalline cone. Colors denote opsin mRNA expression deduced from (**CD**) and (**F**, **HK**).

**Figure 4:**
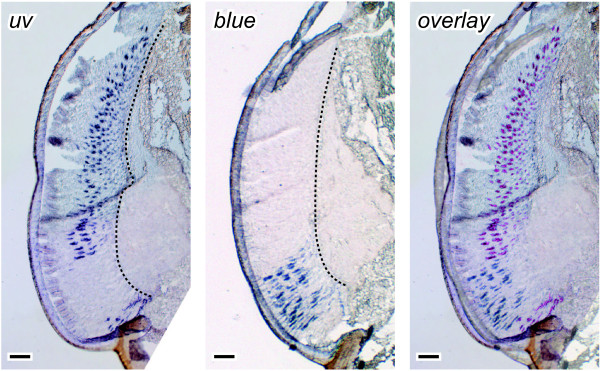
**Localization of *uv *and *blue *mRNA in the pigmented part of the compound eye.** Consecutive dorso-ventral sections through the retina of an adult female were hybridized with antisense *uv* and *blue* riboprobes. The right panel shows an overlay of the two pictures to the left with the *uv* signals re-colored in purple. UV and blue opsin are transcribed in non-overlapping eye regions and at least partly at different levels of the ommatidia. The *blue* labeling is most intense distally, whereas *uv* is generally detected further proximally. Up corresponds to dorsal in all panels. Broken line = basement membrane, scale bars = 100 μm.

Transcripts of three of the four cricket opsins were detected in the compound eyes: *in situ* probes for *greenB* labeled the retina outside the DRA (Figure 3A), probes for *uv* labeled photoreceptors in all eye regions except for an area in the ventral half (Figure 3B) and those for *blue* labeled the DRA and receptors in a restricted ventral band of ommatidia (Figure 3C). Consecutive cryostat sections hybridized with antisense *uv* and *blue* riboprobes revealed that the area devoid of *uv* expression coincides with the ventral *blue* band (Figure 4). Thus, the retina of the cricket compound eye is spectrally divided into three parts: the polarization-sensitive DRA expressing Blue- and UV-opsin, a newly-discovered ventral band expressing Blue- and Green-opsin, and the remainder of the compound eye expressing UV- and Green-opsin. This expression pattern was found in the retina of adults and larvae of both sexes.

#### UV and blue receptors in the DRA

In an attempt to relate opsin expression to specific photoreceptors, we number the retinula cells of the cricket ommatidium according to Burghause [45]. In the DRA, cells 3 and 4 do not form microvilli although they extend from the crystalline cone all the way down to the basement membrane (Figure 3L). The five receptors 1, 2, 5, 6 and 7 contribute to the rhabdom along its entire length, while the short receptor 8 joins in only proximally. *In situ* hybridizations suggest that all functional receptors in the DRA, except for the proximal cell 8, transcribe *blue* (Figures 3A-D, F, H). A proximal *uv* staining in the DRA (arrowheads in Figures 3B, F) can probably be attributed to cell 8, an apparently fully developed photoreceptor despite its small size [51].

#### Blue and green receptors in a ventral band

According to previous anatomical studies, the rhabdomeres of all 8 photoreceptors contribute to the rhabdom in the pigmented part of the cricket retina. Cells 1, 3, 5 and 7 begin most distally (Figure 3L) and are then joined by receptors 2, 4 and 6 below the crystalline cone. Retinula cell 8 and its rhabdomere are developed in the proximal half of each ommatidium only [45,76]. Assuming that the structure of the ommatidia is the same everywhere in the retina except for the DRA, we infer from our data that at least one of the four receptors 1, 3, 5 and 7 transcribes *blue* in the ventral blue band. This is because *in situ* hybridizations show that the *blue* staining reaches up to the crystalline cone (Figures 3C, E and 4). The remaining retinula cells are labeled by probes for *greenB* (Figures 3A, H) and are thus most likely green receptors.

#### UV and green receptors in the main retina

According to our data, receptors 1, 3, 5 and 7 are green-sensitive in the central retina (Figure 3L), since distal cross-sections reveal that all four retinula cells surrounding the tip of the crystalline cone transcribe *greenB* (Figure 3I). Proximal to the crystalline cone, the *greenB* labeling becomes weaker (Figure 3H, J) and cells stained for *uv* can be detected (Figure 3K). This indicates that at least one of the three receptors 2, 4 and 6 is UV-sensitive, while the others are either also UV-sensitive or green-sensitive (see striped cell bodies in Figure 3L). Opsin expression in the proximal cell 8 could not be clarified, as we were unable to identify the small receptor in this part of the retina. Our data suggest that the receptor arrangement in the periphery of the pigmented eye region can also deviate from the pattern described above. For example, in the ommatidia directly adjacent to the DRA and in those ventral to the ventral band (arrows in Figure 3F and G), we sometimes observed that the *uv* expressing cells extended further towards the crystalline cone than in the ommatidia of the central retina.

### Cricket ocelli

#### The ocelli are green- and UV-sensitive

**Figure 5:**
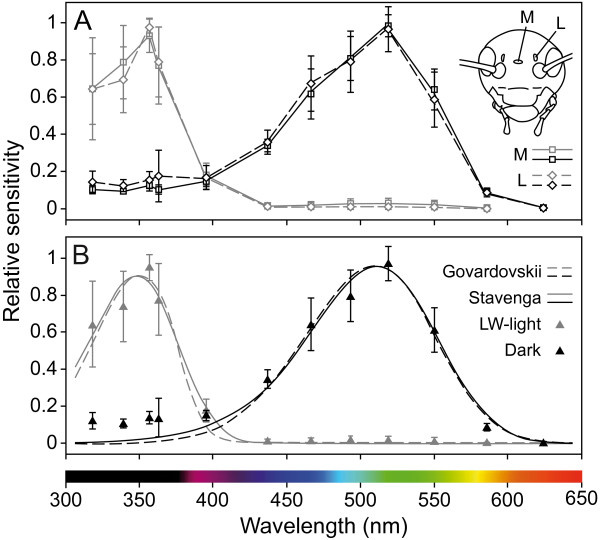
**Spectral sensitivity of the ocelli derived from ERG recordings.** (**A**) Mean spectral sensitivity (± standard deviation) of the median (M, solid lines, open squares, n = 12) and the left ocellus (L, broken lines, open diamonds, n = 12) under dark-adaptation (black) and light-adaptation with bright long-wavelength (LW) illumination (grey). The similarity between the two ocelli in peak sensitivity and in the shape of the curves is evident. (**B**) Pooled spectral sensitivity data of both ocelli (mean value ± standard deviation, n = 24) under dark-adaptation (black triangles) and LW-adaptation (grey triangles) are well fitted by templates for the α-band of an 11-cis retinal visual pigment. The template formulae developed by Stavenga (solid lines) and Govardovskii (broken lines) yield peak absorbances at 511 nm and 510 nm for the dark-adapted state and 348 nm and 351 nm for the LW-adapted state, respectively.

We measured the spectral sensitivities of the median and the left ocellus in 12 adult *G. bimaculatus*, 5 males and 7 females, by ERG recordings. All animals were first tested under dark-adaptation. To explore the existence of SW-receptors, which may be masked by more abundant LW-receptors, we then tested the animals under chromatic adaptation with bright LW-illumination. We found neither a significant difference between individual crickets (*χ*^2^ = 2.7, *df* = 1, *P* = 0.1003 for dark-adaptation, *χ*^2^ = 0.6, *df* = 1, *P* = 0.4385 for LW-adaptation) nor between the median and the left ocellus (*F*_1,63.3_ = 2.01; *P* = 0.1613 for dark-adaptation, *F*_1,44.6_ = 0.50; *P* = 0.4823 for LW-adaptation). Plotting the values for both ocelli on the same graph shows the similarity of their sensitivity curves (Figure 5A). Sensitivities were therefore pooled before templates for the α-band of an 11-cis retinal visual pigment [72] were fitted to the data using least squares regression (Figure 5B). The template formulae developed by Stavenga [73] and Govardovskii [74] produce equal correlations (0.99 for dark-adaptation and 0.97 for LW-adaptation; correlation was calculated as 1 - mean square error). For the dark-adapted state the Stavenga and the Govardovskii templates give peak absorbances at λ_max_ = 511 and 510 nm and for the LW-adapted state at λ_max_ = 348 and 351 nm, respectively. Thus, cricket ocelli have both green- and UV-receptors.

#### Two ocellus-specific opsins

**Figure 6:**
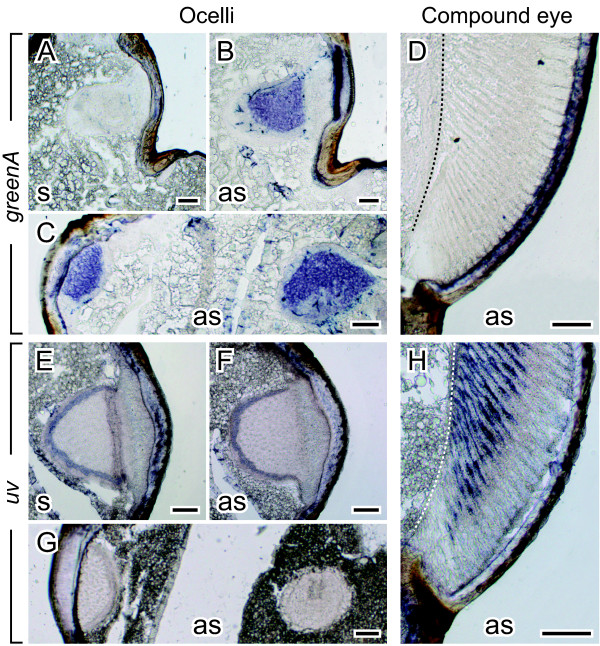
**Localization of *greenA *and *uv *mRNA in the ocelli and compound eyes.** Transverse sections through the head of an adult male were hybridized with sense (s) and antisense (as) riboprobes. (**A**-**B**, **E**-**F**) Right ocellus sectioned longitudinally. (**C**, **G**) Left ocellus (to the left) sectioned longitudinally and median ocellus (to the right) sectioned tangentially. (**D**, **H**) Ventral part of the compound eye sectioned longitudinally. Whereas *greenA* is transcribed in all three ocelli (**B**-**C**), it is absent from the retina of the compound eyes (**D**). *uv*, on the other hand, is clearly detectable in the compound eyes (**H**) but cannot be found in the ocelli (**F**-**G**). Up corresponds to dorsal in all panels. Broken line = basement membrane, scale bars = 100 μm.

None of the three opsin transcripts expressed in the compound eyes (i.e. *greenB*, *blue* and *uv*) could be found in the ocelli (Figure 6E-H and data not shown). Transcripts of *greenA*, on the other hand, were detected in the median as well as in both lateral ocelli but not in the compound eyes (Figure 6A-D). GreenA groups in the insect LW opsin clade of the molecular phylogenetic tree (Figure 2). It is therefore most likely that GreenA belongs to the green-sensitive visual pigment (λ_max_ ≈ 511 nm) that was discovered in the ocelli by ERG recordings (Figure 5). Selective depression of LW sensitivity by chromatic adaptation revealed the existence of a second ocellar pigment maximally absorbing in the UV spectral range (λ_max_ ≈ 350 nm, Figure 5). However, the opsin that forms the UV-sensitive visual pigment found in the compound eyes is not expressed in the ocelli (Figure 6E-H). Thus, there must be an ad-ditional, yet unknown, ocellus-specific opsin in the cricket *G. bimaculatus*.

## Discussion

### Opsin expression in cricket compound eyes

Our data confirm the existence of the three spectral receptor types that have previously been identified in the cricket compound eye by intracellular recordings [53,54]. Beyond that, our results provide a fine-scaled image of the distribution of spectral sensitivities across the retina. We demonstrate that the eyes of the cricket *G. bimaculatus*, which represents a comparatively early-branching insect lineage, are regionalized in a rather complex pattern. However, we have to bear in mind that our *in situ* hybridizations detect mRNA transcripts. In most cases, the available physiological data support the assumption that these transcripts are translated into proteins, but for the *uv*-expressing cells in the DRA and the *blue*-expressing cells in the ventral band, a physiological verification is missing.

#### Dorsal rim area (DRA)

By intracellular recordings, only blue receptors were found in the DRA of *G. bimaculatus* and *G. campestris*[53,54]. However, the spatial pattern of opsin mRNA expression suggests that the long photoreceptors are blue-sensitive, while the proximal cell 8 is UV-sensitive (Figure 3). The latter is rather short [45] and has therefore probably been missed by electrophysiological investigations. Intracellular recordings from UV receptors in the DRA of the desert locust *Schistocerca gregaria*, a related, orthopteroid insect species, support this assumption [77]. Assuming that the distribution of spectral receptor types is the same in the DRAs of locusts and crickets, it seems to be easier to record from the UV-cell 8 in *S. gregaria*, because it is a long receptor that begins at the same distal level as all other retinula cells [78]. Another interesting difference between *S. gregaria* and *G. bimaculatus* is that receptors 3 and 4 still contribute a few microvilli to the rhabdom in the locust DRA [78], whereas microvilli are completely missing in the corresponding cells of the cricket [45], suggesting that opsin expression might be lost. Considering the different degrees of reduction of retinula cells 3, 4 and 8, one can hypothesize that the DRA of *S. gregaria* and *G. bimaculatus* constitute two different stages in the specialization of the orthopteran DRA for monochromatic polarization vision.

#### Ventral band

Previous intracellular recordings identified UV receptors only in the dorsal region of the pigmented part of the cricket compound eye, and no blue receptors have ever been recorded outside the DRA [53,54]. Our results, in contrast, show that UV opsin transcripts are found everywhere in the pigmented part of the eye, except for a restricted ventral band in which the same blue opsin transcripts are expressed as in the DRA (Figures 3 and 4). The disparity between electrophysiological and molecular findings might be explained by the fact that only few cells have been recorded in the ventral retina. According to our data, both blue and UV receptors occur together with green receptors in the pigmented part of the eye. As green receptors are much more abundant, it is statistically more likely to record from them.

Since no spectral sensitivity measurements of blue receptors outside the DRA exist and since the resolution of our *in situ* hybridizations in the ventral band does not allow us to associate opsin expression to specific retinula cells, we also have to consider that some receptors in the ventral band may co-express green and blue opsin. Co-expression of opsins in insect photoreceptors exists [8,79-82], but it is more unusual than the expression of one opsin per receptor.

The function of the ventral band, which is present in adults and larvae of both sexes, remains unclear. Regionalization, be it by gradual changes in the number and frequency of receptor types or by confined, principally different parts of the eye, is a common property of the insect visual system [83]. However, few studies describe band-like specializations in the ventral half of the compound eye. One example can be found in the backswimmer *Notonecta glauca*. Within its polarization-sensitive ventral eye region is a band of ommatidia that differ from the rest by the orientation of their central rhabdomeres [84]. This retinal band coincides with a high-acuity zone directed towards the space just above the water when the animal is hanging upside-down under the surface [85]. Its function might therefore be related to prey detection. Another example is the mosquito *Aedes aegypti,* in which the R7 cell switches from the expression of UV opsin to the expression of a longer wavelength opsin in a defined ventral band [86]. It has been speculated that this horizontal structure is used for flight stabilization. While cricket larvae lack wings, adult crickets can fly, although they do not fly as habitually as mosquitoes [87-89]. Thus, flight stabilization is a valid hypothesis, but it needs to be tested by suitable experiments before further conclusions can be drawn. Yet another possibility is that the ventral band in *G. bimaculatus* is specialized to detect linear polarized light in the ventral field of view, such as that generated by reflections from bodies of water. We are not aware of any behavioral observations of crickets that could substantiate this argument, but migrating desert locusts have been shown to avoid flying over surfaces with strongly polarized reflections [90]. In addition, electrophysiological studies in *S. gregaria* revealed polarization-sensitive neurons in the brain that apparently received input from eye regions ventral to the DRA [91-93].

#### Photoreceptor arrangement within ommatidia

The photoreceptor arrangement in the ventral band of the cricket corresponds to the pattern typically found in insects: shorter wavelength receptors contribute to the rhabdom distal to LW receptors (reviewed by [94]). Surprisingly, this is not the case in the remainder of the cricket compound eye. It is not the UV receptors but the green- or blue-sensitive cells that contribute to the rhabdom at the most distal level. Since green and blue visual pigments have their β-absorption peak in the UV, such an arrangement leads to a comparatively broad spectral sensitivity function of the distal receptors, while it reduces the absolute sensitivity of the proximal UV receptors. Calculations have shown that these effects are rather small, even if the distal layer constitutes two thirds of the total length of the rhabdom [95]. Nevertheless, the proximal position of UV receptors in insect ommatidia is unusual according to the present state of knowledge and still requires an explanation.

*In situ* hybridizations detect *blue* at a more distal level in the ventral band than *uv* in the central retina (Figure 4). Provided that the structure of the ommatidia is indeed invariable in the pigmented part of the eye, this implies that Blue does not simply replace UV expression. In other words, in the ventral band *blue* is not transcribed in the same retinula cells as *uv* in the central retina. To our knowledge, the photoreceptor arrangement in the ventral part of the cricket compound eye has not been studied anatomically. It is therefore also possible that Blue replaces UV in the receptors 2, 4 or 6, and that these receptors reach more distal levels in the ommatidia of the ventral band than in the ommatidia of the central retina. Even within the main, UV-green-sensitive region, the ommatidial structure might vary, as UV expressing cells can extend further towards the crystalline cone in some locations in the eye (e.g. adjacent to the DRA or ventral from the ventral band, arrows in Figures 3F and G). Apparently, retinal heterogeneity in the cricket compound eye is more complex than previously assumed and worth further investigations. In particular in the ventral retina, different receptor types have to be characterized by electrophysiological recordings combined with dye injections and anatomical studies to complement our knowledge of the retinal mosaic in the cricket.

### Spectral sensitivity and opsin expression in the ocelli

The ocelli of most insect species show sensitivity maxima in both the UV and the blue-green spectral range (dragonflies [96,97], mantis [98], locust [99], bumblebee [100], honeybee [101], moths [102-104], flies [3,105-107]). According to our ERG data, this also applies to the two-spotted cricket *G. bimaculatus*, even though we did not detect transcripts of *uv* in its ocelli. Does that point towards a single spectral mechanism as is present in flies? Flies achieve ocellar UV-sensitivity by a sensitizing pigment that transfers energy to an LW visual pigment (*Musca*[105]; *Calliphora*[105,106]; *Drosophila*[3,107]). We can rule out this possibility, since LW adaptation resulted in selective depression of green-sensitivity, unmasking a weaker UV-sensitivity. Cricket ocelli clearly exhibit two independent spectral mechanisms, as do dragonfly, honeybee and moth ocelli [96,101-104]. While we could identify GreenA opsin as part of the ocellar LW visual pigment in the cricket *G. bimaculatus*, the UV opsin forming the SW visual pigment is still unknown. It is certainly not the one expressed in the compound eyes, contrary to what has been found in other insects [5,108]. Apart from our expression data (Figure 6), the spectral sensitivity of the ocellar UV pigment in *G. bimaculatus* (λ_max_ ~ 350 nm) points towards a different opsin, since none of the pigments in the compound eye yielded a similar λ_max_[54]. Interestingly, ERG recordings under both dark and LW adaptation conditions revealed only green-sensitive receptors (λ_max_ = 520 nm) in the ocelli of the sand field cricket *Gryllus firmus*[109], the closest relative of *G. bimaculatus* studied so far. A close inspection of more cricket species is necessary to clarify the prevalence of ocellar UV sensitivity in the family Gryllidae.

### Differential opsin expression in ocelli and compound eyes

Unlike crickets, honeybees express the same UV opsin (AmUVop) in ocelli and compound eyes [5]. This has also been shown for bumblebees [108]. The LW opsins, in contrast, differ between ocelli and compound eyes in all insect species investigated so far. GreenA of the cricket *G. bimaculatus*, AmLop2 of the honeybee *Apis mellifera*[5] and Rh2 of the fruit fly *Drosophila melanogaster*[6] are ocellus-specific (Figure 2). All three opsins are phylogenetically placed within the insect LW opsin clade and seem to be the result of gene duplications that occurred independently in several insect lineages. Rh2, the ocellus-specific opsin of *D. melanogaster*, for example, is most closely related to Rh1, an opsin expressed in the *Drosophila* compound eye (Figure 2). Rh1 and Rh2 orthologs were identified in species from the two major subgenera of *Drosophila* suggesting that the respective gene duplication predates the genus *Drosophila*[110]. However, Rh1-Rh2 orthologs are absent from the genome of the mosquito *Anopheles gambiae*, another dipteran species [5]. Therefore, the duplication event that gave rise to the Rh1 and Rh2 subclades most likely took place within the dipteran lineage after the ancestors of mosquitoes (nematoceran Diptera) and flies (brachyceran Diptera) had diverged about 250 mya [20]. The ocellus-specific LW opsins of the honeybee *A. mellifera* (Hymenoptera) and of the two-spotted cricket *G. bimaculatus* (Orthoptera) show a different branching pattern. They separate from the LW opsins expressed in insect compound eyes at rather basal positions (Figure 2), but do not form a monophyletic clade. This suggests that they go back to two independent duplication events, one in the lineage leading to bees and one in the lineage leading to crickets. Sequence data on ocellar opsins of other insect species that could help to narrow down the time interval in which these events took place are lacking. There is, however, evidence that all insect ocellar opsins known so far date from duplication events that are younger than the divergence of hexapods and branchiopod crustaceans in the Late Silurian about 400 Mya ago [20,111]. Branchiopods are considered a sister group of hexapods [112], with the crustacean Nauplius eye being homologous to insect ocelli [22,23]. Nevertheless, neither the opsins found in the tadpole shrimp *Triops granarius* (Figure 2; [10]), nor any opsin encoding sequence in the genome of the water flea *Daphnia pulex*, (another branchiopod crustacean) groups with insect ocellar opsins [9]. Chelicerate opsins isolated from the principal eyes of jumping spiders [14] or the median eyes of the horseshoe crab [15] - visual organs presumably homologous to insect ocelli [22,23] - do not cluster with insect ocellar opsins either (Figure 2). Instead, they separate before the insect lineages diversify.

### Implications for the evolutionary origins of ocelli and compound eyes

Why does the differential expression of opsins in insect compound eyes and ocelli seem to be such a recent phenomenon? If the evolutionary divergence of both eye types dates back to the first euarthropods, more than 500 mya [25], was there no need for spectral specialization earlier? We might hypothesize that the different functions of ocelli (or homologous single-lens organs) and compound eyes demand different spectral sensitivities, which could be difficult to achieve with the same opsins. Thus, while neither refuting the phyloge-netic homology of arthropod ocelli nor the one of compound eyes, our results better suit the theory proposed by Oakley, Plachetzki and Rivera [113], which claims that common switching between both eye types occurred in arthropods. More specifically, our data are consistent with the hypothesis that a hexapod ancestor of extant insects only had one type of visual organ from which the other type was regained by a morphological furcation event.

No matter whether we assume that all arthropod ocelli are phylogenetically homologous or whether we limit this assumption to the ocelli of hexapods or the ones of insects, a gap between the time of tissue furcation and opsin gene duplication remains. Similar findings were made in a previous study on arthropod ocular structures [113]. We are thus adding another case to what might be a general phenomenon, at least in arthropods: opsin gene duplication and expression specialization are recent compared to the origins of the ocular structures themselves.

## Conclusions

In this paper, we present the first extensive characterization of visual opsins in a hemimetabolous insect, the two-spotted cricket *Gryllus bimaculatus*, which belongs to a rather early-branching insect lineage. Eye development in Hemimetabola is not as evolutionary derived as in Holometabola and more typical for arthropods in general. Our results thus provide a better basis for in-depth comparisons between the eyes of insects and those of non-hexapod arthropods such as crustaceans and chelicerates.

Interestingly, opsin expression in the two-spotted cricket is more complex than expected. The compound eyes are partitioned into three clearly defined regions, which differ in the distribution of spectral receptor types: (1) The DRA, specialized to detect skylight polarization, contains not only blue receptors, but also expresses UV opsin in a small, proximal retinula cell. (2) Blue opsin is additionally found outside the DRA. It occurs together with green receptors in a newly-discovered horizontal band of ommatidia in the ventral half of the eye. (3) The remainder of the eye contains UV and green receptors. In the DRA and in the central retina, the receptor arrangement within an ommatidium is atypical, since UV receptors contribute to the rhabdom proximal to longer wavelength receptors and not *vice versa*, as in other insect species [94]. This illustrates that generalizations from studies on the eyes of holometabolous insects may not hold for all insects, and that more surprises are to be expected if we extend our knowledge to understudied insect groups.

Finally, we provide evidence that the opsins expressed in the cricket ocelli differ from those expressed in the compound eyes. Phylogenetic analyses place all insect ocellar opsins in the visual opsin clades of insects together with opsins expressed in the compound eyes. The branching pattern of ocellar opsins differs for different insect taxa. From that, we conclude that gene duplications, which permitted differential opsin expression in insect ocelli and compound eyes, occurred independently in several insect lineages and are recent compared to the origin of the eyes themselves.

## Abbreviations

DRA: dorsal rim area; ERG: electroretinogram; *Gb*: *Gryllus bimaculatus*; G90: amino acid residue glycine at position 90 of bovine rhodopsin; LW: long-wavelength; MW: middle-wavelength; mya: million years ago; SW: short-wavelength; λ: wavelength; λ_max_: wavelength of maximal sensitivity.

## Competing interests

The authors declare that they have no competing financial or non-financial interests in relation to the work described in this manuscript.

## Authors' contributions

MJH cloned the opsin genes, carried out expression analyses, performed electroretinogram recordings, evaluated the data, finalized the figures and wrote the manuscript. KD performed expression analyses. MK carried out electroretinogram recordings and prepared the figures. TL conceived the electrophysiological part of the study and revised the manuscript. MG designed the primers, annotated the *Gb* opsin genes, performed database searches and the phylogenetic analysis and drafted part of the manuscript. All authors have read and approved the final manuscript.

## Supplementary Material

Additional file 1 **Table 1.**Primers used for amplification of * Gryllus bimaculatus * opsins. Click here for file

Additional file 2 **Table 2.**References for data presented in Figures 2 and S1. Click here for file

Additional file 3 FASTA file containing the amino acid sequences of all opsins included in our phylogenetic analyses. Click here for file

Additional file 4 **Figure S1.**Alternative phylogenetic trees of insect visual opsins reconstructed by the Maximum likelihood approach. Click here for file

## References

[B1] Porter ML Blasic JR Bok MJ Cameron EG Pringle T Cronin TW Robinson PR Shedding new light on opsin evolution Proc Biol Sci 2012 279 3 14 10.1098/rspb.2011.1819 22012981 PMC3223661

[B2] Briscoe A Chittka L The evolution of color vision in insects Annu Rev Entomol 2001 46 471 510 10.1146/annurev.ento.46.1.471 11112177

[B3] Hu KG Reichert H Stark WS Electrophysiological characterization of Drosophila ocelli J Comp Physiol 1978 126 15 24 10.1007/BF01342646

[B4] Briscoe AD Six opsins from the butterfly Papilio glaucus: Molecular phylogenetic evidence for paralogous origins of red-sensitive visual pigments in insects J Mol Evol 2000 51 110 121 10948267 10.1007/s002390010071

[B5] Velarde RA Sauer CD Walden KKO Fahrbach SE Robertson HM Pteropsin: A vertebrate-like non-visual opsin expressed in the honey bee brain Insect Biochem Mol Biol 2005 35 1367 1377 10.1016/j.ibmb.2005.09.001 16291092

[B6] Pollock JA Benzer S Transcript localization of four opsin genes in the three visual organs of Drosophila; RH2 is ocellus specific Nature 1988 333 779 782 10.1038/333779a0 2968518

[B7] Wakakuwa M Stavenga DG Arikawa K Spectral organization of ommatidia in flower-visiting insects Photochem Photobiol 2007 83 27 34 10.1562/2006-03-03-IR-831 16930092

[B8] Jackowska M Bao R Liu Z McDonald EC Cook TA Friedrich M Genomic and gene regulatory signatures of cryptozoic adaptation: Loss of blue sensitive photoreceptors through expansion of long wavelength-opsin expression in the red flour beetle Tribolium castaneum Front Zool 2007 4 24 10.1186/1742-9994-4-24 18154648 PMC2254409

[B9] Colbourne J Pfrender M Gilbert D Thomas W Tucker A Oakley T Tokishita S Aerts A Arnold G Basu M Bauer D Caceres C Carmel L Casola C Choi J Detter J Dong Q Dusheyko S Eads B Frohlich T Geiler-Samerotte K Gerlach D Hatcher P Jogdeo S Krijgsveld J Kriventseva E Kultz D Laforsch C Lindquist E Lopez J The ecoresponsive genome of Daphnia pulex Science 2011 331 555 561 10.1126/science.1197761 21292972 PMC3529199

[B10] Kashiyama K Seki T Numata H Goto SG Molecular characterization of visual pigments in Branchiopoda and the evolution of opsins in Arthropoda Mol Biol Evol 2009 26 299 311 10.1093/molbev/msn251 18984904

[B11] Oakley TH Huber DR Differential expression of duplicated opsin genes in two eye types of ostracod crustaceans J Mol Evol 2004 59 239 249 10.1007/s00239-004-2618-7 15486697

[B12] Porter ML Bok MJ Robinson PR Cronin TW Molecular diversity of visual pigments in Stomatopoda (Crustacea) Vis Neurosci 2009 26 255 265 10.1017/S0952523809090129 19534844

[B13] Rajkumar P Rollmann SM Cook TA Layne JE Molecular evidence for color discrimination in the Atlantic sand fiddler crab, Uca pugilator J Exp Biol 2010 213 4240 4248 10.1242/jeb.051011 21113005 PMC4074279

[B14] Koyanagi M Nagata T Katoh K Yamashita S Tokunaga F Molecular evolution of arthropod color vision deduced from multiple opsin genes of jumping spiders J Mol Evol 2008 66 130 137 10.1007/s00239-008-9065-9 18217181

[B15] Smith WC Price DA Greenberg RM Battelle BA Opsins from the lateral eyes and ocelli of the horseshoe crab, Limulus polyphemus Proc Natl Acad Sci USA 1993 90 6150 6154 10.1073/pnas.90.13.6150 8327495 PMC46885

[B16] Dalal JS Jinks RN Cacciatore C Greenberg RM Battelle BA Limulus opsins: Diurnal regulation of expression Vis Neurosci 2003 20 523 534 14977331 10.1017/s095252380320506x

[B17] Katti C Kempler K Porter ML Legg A Gonzalez R Garcia-Rivera E Dugger D Battelle BA Opsin co-expression in Limulus photoreceptors: Differential regulation by light and a circadian clock J Exp Biol 2010 213 2589 2601 10.1242/jeb.043869 20639420 PMC2905303

[B18] Mito T Noji S The two-spotted cricket Gryllus bimaculatus: An emerging model for developmental and regeneration studies CSH Protoc 2008 2008 pdb.emo110 10.1101/pdb.emo110 21356736

[B19] Grimaldi D Engel MS Evolution of the Insects 2005 Cambridge: zCambridge University Press

[B20] Gaunt MW Miles MA An insect molecular clock dates the origin of the insects and accords with palaeontological and biogeographic landmarks Mol Biol Evol 2002 19 748 761 10.1093/oxfordjournals.molbev.a004133 11961108

[B21] Homberg U Christensen TA Multisensory processing in the insect brain Methods in Insect Sensory Neuroscience 2004 Boca Raton: CRC Press 3 25

[B22] Bitsch C Bitsch J Koenemann S, Jenner RA, Schram FR Evolution of eye structure and arthropod phylogeny Crustacea and arthropod relationships 2005 New York: CRC Press 185 214

[B23] Paulus HF Gupta AP Eye structure and the monophyly of the Arthropoda Arthropod Phylogeny 1979 New York: Van Nostrand Reinhold Company 299 383

[B24] Paulus HF Phylogeny of the Myriapoda-Crustacea-Insecta: A new attempt using photoreceptor structure J Zoolog Syst Evol Res 2000 38 189 208 10.1046/j.1439-0469.2000.383152.x

[B25] Waloszek D The "Orsten" window - Three-dimensionally preserved Upper Cambrian meiofauna and its contribution to our understanding of the evolution of Arthropoda Paleontological Res 2003 7 71 88 10.2517/prpsj.7.71

[B26] Mayer G Structure and development of onychophoran eyes: What is the ancestral visual organ in arthropods? Arthropod Struct Dev 2006 35 231 245 10.1016/j.asd.2006.06.003 18089073

[B27] Nilsson D-E Kelber A A functional analysis of compound eye evolution Arthropod Struct Dev 2007 36 373 385 10.1016/j.asd.2007.07.003 18089116

[B28] Stavenga DG Eye regionalization and spectral tuning of retinal pigments in insects Trends Neurosci 1992 15 213 218 10.1016/0166-2236(92)90038-A 1378665

[B29] Stavenga DG Kinoshita M Yang EC Arikawa K Retinal regionalization and heterogeneity of butterfly eyes Naturwissenschaften 2001 88 477 481 10.1007/s001140100268 11771477

[B30] Friedrich M Evolution of insect eye development: First insights from fruit fly, grasshopper and flour beetle Integr Comp Biol 2003 43 508 521 10.1093/icb/43.4.508 21680459

[B31] Friedrich M Ancient mechanisms of visual sense organ development based on comparison of the gene networks controlling larval eye, ocellus, and compound eye specification in Drosophila Arthropod Struct Dev 2006 35 357 378 10.1016/j.asd.2006.08.010 18089081

[B32] Friedrich M Opsins and cell fate in the Drosophila Bolwig organ: Tricky lessons in homology inference Bioessays 2008 30 980 993 10.1002/bies.20803 18800378

[B33] Goodman LJ Autrum H Organisation and physiology of the insect dorsal ocellar system Handbook of Sensory Physiology 1981 Volume VII/6C Berlin, Heidelberg, New York: Springer-Verlag 201 286

[B34] Mizunami M Information processing in the insect ocellar system: Comparative approaches to the evolution of visual processing and neural circuits Adv In Insect Phys 1994 25 151 265

[B35] Rence B Lisy M Garves B Quinlan B The role of ocelli in circadian singing rhythms of crickets Physiol Entomol 1988 13 201 212 10.1111/j.1365-3032.1988.tb00924.x

[B36] Nowosielski J Patton R Studies on circadian rhythm of the house cricket, Gryllus domesticus L J Insect Physiol 1963 9 401 410 10.1016/0022-1910(63)90049-0

[B37] Yukizane M Tomioka K Neural pathways involved in mutual interactions between optic lobe circadian pacemakers in the cricket Gryllus bimaculatus J Comp Physiol A 1995 176 601 610 10.1007/BF01021580

[B38] Abe Y Ushirogawa H Tomioka K Circadian locomotor rhythms in the cricket, Gyrllodes sigillatus - I. Localization of the pacemaker and the photoreceptor Zool Sci 1997 14 719 727 10.2108/zsj.14.719 9450385

[B39] Jander R Barry C Phototactic push-pull-coupling between dorsal ocelli and compound eyes in phototropotaxis of locusts and crickets (Saltatoptera - Locusta migratoria and Gryllus bimaculatus) Z Vergl Physiol 1968 57 432 458 10.1007/BF00303067

[B40] Ribi W Warrant E Zeil J The organization of honeybee ocelli: Regional specializations and rhabdom arrangements Arthropod Struct Dev 2011 40 509 520 10.1016/j.asd.2011.06.004 21945450

[B41] Krapp HG Ocelli Curr Biol 2009 19 R435 437 10.1016/j.cub.2009.03.034 19515345

[B42] Pohl R Das postembryonale Wachstum der Retina und die Anatomie von Lamina und Medulla bei Gryllus bimaculatus (De Geer 1773) Zool Jb Anat Volume 117 1988 Jena: VEB Gustav Fischer Verlag 353 393

[B43] Labhart T Keller K Fine-structure and growth of the polarization-sensitive dorsal rim area in the compound eye of larval crickets Naturwissenschaften 1992 79 527 529 10.1007/BF01135777

[B44] Takagi A Kurita K Terasawa T Nakamura T Bando T Moriyama Y Mito T Noji S Ohuchi H Functional analysis of the role of eyes absent and sine oculis in the developing eye of the cricket Gryllus bimaculatus Dev Growth Differ 2012 54 227 240 10.1111/j.1440-169X.2011.01325.x 22348272

[B45] Burghause FMHR Structural specialization in the dorso-frontal region of the cricket compound eye (Orthoptera, Grylloidea) Zool Jahrb Abt Allg Zool Physiol Tiere 1979 83 502 525

[B46] Nilsson D-E Labhart T Meyer E Photoreceptor design and optical properties affecting polarization sensitivity in ants and crickets J Comp Physiol A 1987 161 645 658 10.1007/BF00605006

[B47] Ukhanov K Leertouwer H Gribakin F Stavenga D Dioptrics of the facet lenses in the dorsal rim area of the cricket Gryllus bimaculatus J Comp Physiol A 1996 179 545 552

[B48] Labhart T Meyer E Detectors for polarized skylight in insects: A survey of ommatidial specializations in the dorsal rim area of the compound eye Microsc Res Tech 1999 47 368 379 10.1002/(SICI)1097-0029(19991215)47:6<368::AID-JEMT2>3.0.CO;2-Q 10607378

[B49] Labhart T Meyer E Neural mechanisms in insect navigation: Polarization compass and odometer Curr Opin Neurobiol 2002 12 707 714 10.1016/S0959-4388(02)00384-7 12490263

[B50] Wehner R Labhart T Warrant E, Nilsson DE Polarisation vision Invertebrate Vision 2006 Cambridge, New York, Melbourne, Madrid, Cape Town, Singapore, São Paulo: Cambridge University Press 291 348

[B51] Blum M Labhart T Photoreceptor visual fields, ommatidial array, and receptor axon projections in the polarisation-sensitive dorsal rim area of the cricket compound eye J Comp Physiol A 2000 186 119 128 10.1007/s003590050012 10707310

[B52] Brunner D Labhart T Behavioral evidence for polarization vision in crickets Physiol Entomol 1987 12 1 10 10.1111/j.1365-3032.1987.tb00718.x

[B53] Labhart T Hodel B Valenzuela I The physiology of the cricket's compound eye with particular reference to the anatomically specialized dorsal rim area J Comp Physiol 1984 155 289 296 10.1007/BF00610582

[B54] Zufall F Schmitt M Menzel R Spectral and polarized-light sensitivity of photoreceptors in the compound eye of the cricket (Gryllus bimaculatus) J Comp Physiol A 1989 164 597 608 10.1007/BF00614502 2709343

[B55] Labhart T Petzold J Helbling H Spatial integration in polarization-sensitive interneurones of crickets: A survey of evidence, mechanisms and benefits J Exp Biol 2001 204 2423 2430 11511657 10.1242/jeb.204.14.2423

[B56] Henze MJ Labhart T Haze, clouds and limited sky visibility: Polarotactic orientation of crickets under difficult stimulus conditions J Exp Biol 2007 219 3266 3276 17766304 10.1242/jeb.007831

[B57] Chapman RF The Insects. Structure and Function 1998 Cambridge: Cambridge University Press

[B58] GenBank http://www.ncbi.nlm.nih.gov/Genbank/

[B59] The International Aphid Genomics Consortium Genome sequence of the pea aphid Acyrthosiphon pisum PLoS Biol 2010 8 e1000313 10.1371/journal.pbio.1000313 20186266 PMC2826372

[B60] The Phylogeny.fr platform http://www.phylogeny.fr/version2_cgi/phylogeny.cgi

[B61] Edgar RC MUSCLE: Multiple sequence alignment with high accuracy and high throughput Nucleic Acids Res 2004 32 1792 1797 10.1093/nar/gkh340 15034147 PMC390337

[B62] Castresana J Selection of conserved blocks from multiple alignments for their use in phylogenetic analysis Mol Biol Evol 2000 17 540 552 10.1093/oxfordjournals.molbev.a026334 10742046

[B63] Guindon S Gascuel O A simple, fast, and accurate algorithm to estimate large phylogenies by maximum likelihood Syst Biol 2003 52 696 704 10.1080/10635150390235520 14530136

[B64] Whelan S Goldman N A general empirical model of protein evolution derived from multiple protein families using a maximum-likelihood approach Mol Biol Evol 2001 18 691 699 10.1093/oxfordjournals.molbev.a003851 11319253

[B65] Anisimova M Gascuel O Approximate likelihood-ratio test for branches: A fast, accurate, and powerful alternative Syst Biol 2006 55 539 552 10.1080/10635150600755453 16785212

[B66] Chevenet F Brun C Banuls AL Jacq B Christen R TreeDyn: towards dynamic graphics and annotations for analyses of trees BMC Bioinformatics 2006 7 439 10.1186/1471-2105-7-439 17032440 PMC1615880

[B67] Westerfield M The zebrafish book: A guide for the laboratory use of zebrafish (Danio rerio) 2007 5 Eugene: University of Oregon Press

[B68] Wolfinger R Covariance structure selection in general mixed models Commun Stat Simulat 1993 22 1079 1106 10.1080/03610919308813143

[B69] Wolfinger RD Heterogeneous variance-covariance structures for repeated measures J Agric Biol Envir Stat 1996 1 205 230 10.2307/1400366

[B70] Schaalje GB McBride JB Fellingham GW Adequacy of approximations to distributions of test statistics in complex mixed linear models J Agric Biol Environ Stat 2002 7 512 524 10.1198/108571102726

[B71] Littell RC Milliken GA Stroup WW Wolfinger RD SAS System for mixed models 1996 Cary, NC: SAS Publishing

[B72] Seki T Fujishita S Ito M Matsuoka N Tsukida K Retinoid composition in the compound eyes of insects Exp Biol 1987 47 95 103 3436407

[B73] Stavenga DG Smits RP Hoenders BJ Simple exponential functions describing the absorbency bands of visual pigment spectra Vision Res 1993 33 1011 1017 10.1016/0042-6989(93)90237-Q 8506642

[B74] Govardovskii VI Fyhrquist N Reuter T Kuzmin DG Donner K In search of the visual pigment template Vis Neurosci 2000 17 509 528 10.1017/S0952523800174036 11016572

[B75] Salcedo E Zheng L Phistry M Bagg EE Britt SG Molecular basis for ultraviolet vision in invertebrates J Neurosci 2003 23 10873 10878 14645481 10.1523/JNEUROSCI.23-34-10873.2003 PMC2819302

[B76] Sakura M Takasuga K Watanabe M Eguchi E Diurnal and circadian rhythm in compound eye of cricket (Gryllus bimaculatus): Changes in structure and photon capture efficiency Zoolog Sci 2003 20 833 840 10.2108/zsj.20.833 12867711

[B77] Eggers A Gewecke M Wiese K, Gribakin FG, Popov AV, Renninger G The dorsal rim area of the compound eye and polarization vision in the desert locust (Schistocerca gregaria) Sensory Systems of Arthropods 1993 Basel: Birkhäuser Verlag 101 109

[B78] Homberg U Paech A Ultrastructure and orientation of ommatidia in the dorsal rim area of the locust compound eye Arthropod Struct Dev 2002 30 271 280 10.1016/S1467-8039(02)00010-5 18088961

[B79] Kitamoto J Sakamoto K Ozaki K Mishina Y Arikawa K Two visual pigments in a single photoreceptor cell: Identification and histological localization of three mRNAs encoding visual pigment opsins in the retina of the butterfly Papilio xuthus J Exp Biol 1998 201 1255 1261 9547302 10.1242/jeb.201.9.1255

[B80] Arikawa K Mizuno S Kinoshita M Stavenga DG Coexpression of two visual pigments in a photoreceptor causes an abnormally broad spectral sensitivity in the eye of the butterfly Papilio xuthus J Neurosci 2003 23 4527 4532 12805293 10.1523/JNEUROSCI.23-11-04527.2003 PMC6740815

[B81] Sison-Mangus MP Bernard GD Lampel J Briscoe AD Beauty in the eye of the beholder: The two blue opsins of lycaenid butterflies and the opsin gene-driven evolution of sexually dimorphic eyes J Exp Biol 2006 209 3079 3090 10.1242/jeb.02360 16888057

[B82] Mazzoni EO Celik A Wernet MF Vasiliauskas D Johnston RJ Cook TA Pichaud F Desplan C Iroquois complex genes induce co-expression of rhodopsins in Drosophila PLoS Biol 2008 6 e97 10.1371/journal.pbio.0060097 18433293 PMC2323304

[B83] Kelber A Warrant E, Nilsson D-E Invertebrate colour vision Invertebrate Vision 2006 Cambridge: Cambridge University Press 250 290

[B84] Schwind R Zonation of the optical environment and zonation in the rhabdom structure within the eye of the backswimmer, Notonecta glauca Cell Tissue Res 1983 232 53 63 6883440 10.1007/BF00222373

[B85] Schwind R Geometrical-optics of the notonecta eye - adaptations to optical environment and way of life J Comp Physiol 1980 140 59 68 10.1007/BF00613748

[B86] Hu X England JH Lani AC Tung JJ Ward NJ Adams SM Barber KA Whaley MA O'Tousa JE Patterned rhodopsin expression in R7 photoreceptors of mosquito retina: Implications for species-specific behavior J Comp Neurol 2009 516 334 342 10.1002/cne.22114 19637310 PMC2845463

[B87] Ragge DR An unusual case of mass migration by flight in Gryllus bimaculatus DeGeer Orthoptera Gryllidae BIFAN, Series A 1972 34 869 878

[B88] Tanaka S Tanaka K Yasuhara Y Nakahara Y Katagiri C Flight activity, flight fuels and lipophorins in a cricket, Gryllus bimaculatus Entomological Science 1999 2 457 465

[B89] Lorenz MW Oogenesis-flight syndrome in crickets: Age-dependent egg production, flight performance, and biochemical composition of the flight muscles in adult female Gryllus bimaculatus J Insect Physiol 2007 53 819 832 10.1016/j.jinsphys.2007.03.011 17490675

[B90] Shashar N Sabbah S Aharoni N Migrating locusts can detect polarized reflections to avoid flying over the sea Biol Lett 2005 1 472 475 10.1098/rsbl.2005.0334 17148236 PMC1626356

[B91] el Jundi B Pfeiffer K Homberg U A Distinct Layer of the Medulla Integrates Sky Compass Signals in the Brain of an Insect PLoS One 2011 6 e27855 10.1371/journal.pone.0027855 22114712 PMC3218074

[B92] Heinze S Gotthardt S Homberg U Transformation of polarized light information in the central complex of the locust J Neurosci 2009 29 11783 11793 10.1523/JNEUROSCI.1870-09.2009 19776265 PMC6666666

[B93] Träger U Homberg U Polarization-sensitive descending neurons in the locust: Connecting the brain to thoracic ganglia J Neurosci 2011 31 2238 2247 10.1523/JNEUROSCI.3624-10.2011 21307260 PMC6633037

[B94] Friedrich M Wood EJ Wu M Developmental evolution of the insect retina: insights from standardized numbering of homologous photoreceptors J Exp Zool B Mol Dev Evol 2011 316 484 499 21796775 10.1002/jez.b.21424

[B95] Warrant E Kelber A Frederiksen R North G, Greenspan RJ Ommatidial adaptations for spatial, spectral, and polarization vision in arthropods Invertebrate neurobiology 2007 New York: Cold Spring Harbor Laboratory Press 123 154

[B96] Chappell RL DeVoe RD Action spectra and chromatic mechanisms of cells in the median ocelli of dragonflies J Gen Physiol 1975 65 399 419 10.1085/jgp.65.4.399 1151320 PMC2214924

[B97] Ruck P The components of the visual system of a dragonfly J Gen Psychol 1965 49 289 307 10.1085/jgp.49.2.289 PMC2195487 19873565

[B98] Sontag C Spectral sensitivity studies on the visual system of the praying mantis, Tenodera sinensis J Gen Physiol 1971 57 93 112 10.1085/jgp.57.1.93 5539340 PMC2203092

[B99] Wilson M Functional organization of locust ocelli J Comp Physiol 1978 124 297 316 10.1007/BF00661380

[B100] Meyer-Rochow VB Electrophysiologically determined spectral efficiencies of the compound eye and median ocellus in the bumblebee Bombus hortorum tarhakimalainen (Hymenoptera, Insecta) J Comp Physiol 1980 139 261 266 10.1007/BF00610457

[B101] Goldsmith TH Ruck PR The spectral sensitivities of the dorsal ocelli of cockroaches and honeybees; an electrophysiological study J Gen Physiol 1958 41 1171 1185 10.1085/jgp.41.6.1171 13563806 PMC2194880

[B102] Eaton JL Spectral sensitivity of the ocelli of the adult cabbage-looper moth, Trichoplusia ni J Comp Physiol 1976 109 17 24 10.1007/BF00663432

[B103] Pappas LG Eaton JL The internal ocellus of Manduca sexta: Electroretinogram and spectral sensitivity J Insect Physiol 1977 23 1355 1358 10.1016/0022-1910(77)90157-3

[B104] Yamazaki S Yamashita S Efferent control in the ocellus of a noctuid moth J Comp Physiol A 1991 169 647 652

[B105] Kirschfeld K Feiler R Vogt K Evidence for a sensitizing pigment in the ocellar photoreceptors of the fly (Musca, Calliphora) J Comp Physiol A 1988 163 421 423 10.1007/BF00604896

[B106] Kirschfeld K Lutz B Spectral sensitivity of ocelli of Calliphora (Diptera) Z Naturforsch C 1977 32 439 441

[B107] Feiler R Harris WA Kirschfeld K Wehrhahn C Zuker CS Targeted misexpression of a Drosophila opsin gene leads to altered visual function Nature 1988 333 737 741 10.1038/333737a0 2455230

[B108] Spaethe J Briscoe AD Molecular characterization and expression of the UV opsin in bumblebees: three ommatidial subtypes in the retina and a new photoreceptor organ in the lamina J Exp Biol 2005 208 2347 2361 10.1242/jeb.01634 15939775

[B109] Lall AB Trouth CO The spectral sensitivity of the ocellar system in the cricket Gryllus firmus (Orthoptera, Gryllidae) J Insect Physiol 1989 35 805 808 10.1016/0022-1910(89)90094-2

[B110] Carulli JP Chen DM Stark WS Hartl DL Phylogeny and physiology of Drosophila opsins J Mol Evol 1994 38 250 262 8006992 10.1007/BF00176087

[B111] Engel M Grimaldi D New light shed on the oldest insect Nature 2004 427 627 630 10.1038/nature02291 14961119

[B112] Glenner H Thomsen PF Hebsgaard MB Sørensen MV Willerslev E The origin of insects Science 2006 314 1883 1884 10.1126/science.1129844 17185588

[B113] Oakley TH Plachetzki DC Rivera AS Furcation, field-splitting, and the evolutionary origins of novelty in arthropod photoreceptors Arthropod Struct Dev 2007 36 386 400 10.1016/j.asd.2007.08.002 18089117

[B114] Merkel G The effects of temperature and food quality on the larval development of Gryllus bimaculatus (Orthoptera, Gryllidae) Oecologia 1977 30 129 140 10.1007/BF00345416 28309428

