# Supplementary material for: Opsin evolution and expression in Arthropod compound Eyes and Ocelli: Insights from the cricket Gryllus bimaculatus
Source: BMC Evol Biol. 2012 Aug 30;12:163. doi: 10.1186/1471-2148-12-163 (PMC3502269; doi:10.1186/1471-2148-12-163)
Supplement: Additional file 1 — Table 1.Primers used for amplification of Gryllus bimaculatus opsins. [file 1471-2148-12-163-S1.pdf]

Table 1 - Primers used for amplification of *Gryllus bimaculatus* opsins.
Please note that the degenerate primers include mismatches to the coding sequence.

Degenerate primers		Gene-specific primers	
**Label**	**Sequence (5'-3')**	**Label**	**Sequence (5'-3')**
UV_deg_s1	ATWCCNGARCAYTGGC	*UV_s1*	GCGCGGGATGGAGCTC
UV_deg_s2	CCGATCTTYATHTAYAAYAG	*UV_s2*	AAGCAATCAACAGCAAAATC
UV_deg_s3, Blue_deg_s3, GreenA_deg_s3, GreenB_deg_s3	GGCCGTTTYGTNCCNGARGG	*UV_s3*	GGCATATGCTTTCTGTTTG
UV_deg_as1, Blue_deg_as1, GreenA_deg_as1	ACGTTCATYTTYTTNGCYTG	*UV_as1*	CGAAGAGACTTGGCTGAAC
UV_deg_as2, Blue_deg_as4, GreenB_deg_as3	ATCACGCCRTANGGNGTCCA	*UV_as2*	GCTCCTATGCCTGAAATTG
Blue_deg_s1	CAAYGGNATCGTCCTNTGGAT	*Blue_s1*	CCCTCGAGACCAGCATAG
Blue_deg_s2	CGTACACCNTCNAAYATGTT	*Blue_s2*	ATCAAGATGCTGAACAAAAATC
Blue_deg_s4	CAGGCNAARAARATGAAYG	*Blue_s3*	GGTAGCATTCACAATTTTCTTC
Blue_deg_s5	ACGCCGTAYGGNGTNATG	*Blue_as1*	CAAGCAAGGCCTGGTTTAC
Blue_deg_as2	AGTGAYTTNACRTTCATYTT	*Blue_as2*	CCTACTTCCCAACCAATTG
Blue_deg_as3	AGCGTANGGNGTCCANG	*Blue_as3*	CGGATTTCAACAGATTTTTG
GreenA_deg_s1	GTGGCTCNATHTGGACNATG	*Blue_as4*	ACCGACTTTATTTTCTACAATTG
GreenA_deg_s2, GreenB_deg_s2	TGCTAYTAYGARACNTGGG	*GreenA_s1*	CCCCACAGTCAGCCAAAG
GreenA_deg_s4	GTCCCTGARGGNAAYATGAC	*GreenA_s2*	CTACTTGCCCCTTTTTACG
GreenA_deg_as2, GreenB_deg_as1	AGAGAGGCNACRTTCATYTTYTT	*GreenA_s3*	CGAACAGGCAAAGAAAATG
GreenA_deg_as3, GreenB_deg_as2	AACCACAGNGADATNGTCAT	*GreenA_as1*	GCACATCAGTTCTCCTAATACC
GreenB_deg_s1	ATGAACCCNCTNTGGCAYGG	*GreenA_as2*	GCTGTCATATTTCCTTCTGG
		GreenA_as3	ACACCGAGTACACCAAAATG
		GreenA_as4	GTTTCAGGAAAGTACATAAAAAC
		GreenB_s1	GCAGCAGAACCCGAAGTAG
		GreenB_s2	GCGCCTCTCTTCACCATC
		GreenB_s3	GCGAGCAAGCCAAGAAG
		GreenB_as1	CCCAGCACCGAGATCAC
		GreenB_as2	GAAGGCCAAGTTGACTACG
		GreenB_as3	ATAAATTAGAGAGAGGGTGCTG
