# Supplementary material for: Opsin evolution and expression in Arthropod compound Eyes and Ocelli: Insights from the cricket Gryllus bimaculatus
Source: BMC Evol Biol. 2012 Aug 30;12:163. doi: 10.1186/1471-2148-12-163 (PMC3502269; doi:10.1186/1471-2148-12-163)
Supplement: Additional file 2 — Table 2.References for data presented in Figures 2 and S1. [file 1471-2148-12-163-S2.pdf]

Table 2 - References for data presented in Figures 2 and S1.
Accession numbers of sequences retrieved from GenBank, references on opsin expression and the wavelength of peak sensitivity (λ_{max}) of the respective visual pigment. For opsin sequences of *Acyrthosiphon pisum* (pea aphid) see Additional file 3.

Scientific name	Common name	Accession numbers	References on gene expression and λ_{max}
Vanessa cardui	painted lady	VanG: AY613986<br>VanB: AY613987<br>VanUV: AF414074	[1]
Drosophila melanogaster	fruit fly	Rh1: K02315<br>Rh2: M12896<br>Rh3: M17718<br>Rh4: M17730<br>Rh5: U67905<br>Rh6: Z86118<br>Rh7: NM_079311	[2-15]
Apis mellifera	honey bee	AmLop1: U26026<br>AmLop2: BK005515<br>AmBLop: AF004168<br>AmUVop: AF004169<br>Pteropsin: BK005510	[16-21]
Tribolium castaneum	red flour beetle	Green: NM_001162519<br>UV: XM_965251	[22]
Megoura viciae	vetch aphid	LW: AF189714<br>UV_A: AF1897145	[23]
Schistocerca gregaria	desert locust	Lo1: X80071<br>Lo2: X80072	[24]
Gryllus bimaculatus	two-spotted cricket	GreenA: HM363620<br>GreenB: HM363621<br>Blue: HM363622<br>UV: HM363623	[25] and this manuscript
Dianemobius nigrofasciatus	band-legged ground cricket	LWop: FJ232921<br>BLop: AB291232<br>UVop: AB458852	
Sphodromantis spec.	praying mantis	Opsin: X71665	
Triops granarius	tadpole shrimp	RhA: AB293428<br>RhB: AB293429<br>RhC: AB293430<br>RhD: AB293431<br>RhE: AB293432	
Hasarius adansoni	Adanson's house jumper	Rh1: AB251846<br>Rh2: AB251847<br>Rh3: AB251848	
Limulus polyphemus	horseshoe crab	Opsin1: L03781<br>Opsin2: L03782<br>Opsin5: FJ791252	[26-30]
Euprymna scolopes	Hawaiian bobtail squid	Eye opsin: EU34773	
Danio rerio	zebrafish	MelanopsinA: NM_001128761	

1. Briscoe A, Bernard G, Szeto A, Nagy L, White R: **Not all butterfly eyes are created equal: Rhodopsin absorption spectra, molecular identification, and localization of ultraviolet-, blue-, and green-sensitive rhodopsin-encoding mRNAs in the retina of *Vanessa cardui*.** *J Comp Neurol* 2003, **458:**334-349.

2. Bernard GD, Stavenga DG: **Spectral sensitivities of retinular cells measured in intact, living flies by an optical method.** *J Comp Physiol A* 1979, **143:**95-107.

3. Fryxell KJ, Meyerowitz EM: **An opsin gene that is expressed only in the R7 photoreceptor cell of *Drosophila*.** *EMBO J* 1987, **6:**443-451.

4. Montell C, Jones K, Zuker C, Rubin G: **A second opsin gene expressed in the ultraviolet-sensitive R7 photoreceptor cells of *Drosophila melanogaster*.** *J Neurosci* 1987, **7:**1558-1566.

5. Zuker CS, Montell C, Jones K, Laverty T, Rubin GM: **A rhodopsin gene expressed in photoreceptor cell R7 of the *Drosophila* eye: homologies with other signal-transducing molecules.** *J Neurosci* 1987, **7:**1550-1557.

6. Feiler R, Harris WA, Kirschfeld K, Wehrhahn C, Zuker CS: **Targeted misexpression of a *Drosophila* opsin gene leads to altered visual function.** *Nature* 1988, **333:**737-741.

7. Pollock JA, Benzer S: **Transcript localization of four opsin genes in the three visual organs of *Drosophila*; RH2 is ocellus specific.** *Nature* 1988, **333:**779-782.

8. Fortini ME, Rubin GM: **Analysis of cis-acting requirements of the Rh3 and Rh4 genes reveals a bipartite organization to rhodopsin promoters in *Drosophila melanogaster*.** *Genes Dev* 1990, **4:**444-463.

9. Feiler R, Bjornson R, Kirschfeld K, Mismer D, Rubin GM, Smith DP, Socolich M, Zuker CS: **Ectopic expression of ultraviolet-rhodopsins in the blue photoreceptor cells of *Drosophila*: visual physiology and photochemistry of transgenic animals.** *J Neurosci* 1992, **12:**3862-3868.

10. Chou WH, Hall KJ, Wilson DB, Wideman CL, Townson SM, Chadwell LV, Britt SG: **Identification of a novel *Drosophila* opsin reveals specific patterning of the R7 and R8 photoreceptor cells.** *Neuron* 1996, **17:**1101-1115.

11. Huber A, Schulz S, Bentrop J, Groell C, Wolfrum U, Paulsen R: **Molecular cloning of *Drosophila* Rh6 rhodopsin: the visual pigment of a subset of R8 photoreceptor cells.** *FEBS Lett* 1997, **406:**6-10.

12. Papatsenko D, Sheng G, Desplan C: **A new rhodopsin in R8 photoreceptors of *Drosophila*: evidence for coordinate expression with Rh3 in R7 cells.** *Development* 1997, **124:**1665-1673.

13. Salcedo E, Huber A, Henrich S, Chadwell LV, Chou WH, Paulsen R, Britt SG: **Blue- and green-absorbing visual pigments of *Drosophila*: ectopic expression and physiological characterization of the R8 photoreceptor cell-specific Rh5 and Rh6 rhodopsins.** *J Neurosci* 1999, **19:**10716-10726.

14. Mazzoni EO, Celik A, Wernet MF, Vasiliauskas D, Johnston RJ, Cook TA, Pichaud F, Desplan C: **Iroquois complex genes induce co-expression of rhodopsins in *Drosophila*.** *PLoS Biol* 2008, **6:**e97.

15. Hu KG, Reichert H, Stark WS: **Electrophysiological characterization of *Drosophila* ocelli.** *J Comp Physiol* 1978, **126:**15-24.

16. Goldsmith TH, Ruck PR: **The spectral sensitivities of the dorsal ocelli of cockroaches and honeybees; an electrophysiological study.** *J Gen Physiol* 1958, **41:**1171-1185.

17. Menzel R, Blakers M: **Colour receptors in the bee eye - morphology and spectral sensitivity.** *J Comp Physiol A* 1976, **108:**11-33.

18. Peitsch D, Fietz A, Hertel H, de Souza J, Ventura DF, Menzel R: **The spectral input systems of hymenopteran insects and their receptor-based colour vision.** *J Comp Physiol A* 1992, **170:**23-40.

19. Townson SM, Chang BS, Salcedo E, Chadwell LV, Pierce NE, Britt SG: **Honeybee blue- and ultraviolet-sensitive opsins: cloning, heterologous expression in *Drosophila*, and physiological characterization.** *J Neurosci* 1998, **18:**2412-2422.

20. Wakakuwa M, Kurasawa M, Giurfa M, Arikawa K: **Spectral heterogeneity of honeybee ommatidia.** *Naturwissenschaften* 2005, **92:**464-467.

21. Velarde RA, Sauer CD, Walden KKO, Fahrbach SE, Robertson HM: **Pteropsin: A vertebrate-like non-visual opsin expressed in the honey bee brain.** *Insect Biochem Mol Biol* 2005, **35:**1367-1377.

22. Jackowska M, Bao R, Liu Z, McDonald EC, Cook TA, Friedrich M: **Genomic and gene regulatory signatures of cryptozoic adaptation: Loss of blue sensitive photoreceptors through expansion of long wavelength-opsin expression in the red flour beetle *Tribolium castaneum*.** *Front Zool* 2007, **4:**24.

23. Gao N, Foster R, Hardie J: **Two opsin genes from the vetch aphid, *Megoura viciae*.** *Insect Mol Biol* 2000, **9:**197-202.

24. Eggers A, Gewecke M: **The dorsal rim area of the compound eye and polarization vision in the desert locust (*Schistocerca gregaria*).** In *Sensory Systems of Arthropods.* Edited by Wiese K, Gribakin FG, Popov AV, Renninger G. Basel: Birkhäuser Verlag; 1993: 101-109

25. Zufall F, Schmitt M, Menzel R: **Spectral and polarized-light sensitivity of photoreceptors in the compound eye of the cricket (*Gryllus bimaculatus*).** *J Comp Physiol A* 1989, **164:**597-608.

26. Chapman RM, Lall AB: **Electroretinogram characteristics and the spectral mechanisms of the median ocellus and the lateral eye in *Limulus polyphemus*.** *J Gen Physiol* 1967, **50:**2267-2287.

27. Lall AB: **Spectral sensitivity of intracellular responses from visual cells in median ocellus of *Limulus polyphemus*.** *Vision Res* 1970, **10:**905-909.

28. Smith WC, Price DA, Greenberg RM, Battelle BA: **Opsins from the lateral eyes and ocelli of the horseshoe crab, *Limulus polyphemus*.** *Proc Natl Acad Sci USA* 1993, **90:**6150-6154.

29. Dalal JS, Jinks RN, Cacciatore C, Greenberg RM, Battelle BA: ***Limulus* opsins: Diurnal regulation of expression.** *Vis Neurosci* 2003, **20:**523-534.

30. Katti C, Kempler K, Porter ML, Legg A, Gonzalez R, Garcia-Rivera E, Dugger D, Battelle BA: **Opsin co-expression in *Limulus* photoreceptors: differential regulation by light and a circadian clock.** *J Exp Biol* 2010, **213:**2589-2601.
