# Supplementary material for: Opsin evolution and expression in Arthropod compound Eyes and Ocelli: Insights from the cricket Gryllus bimaculatus
Source: BMC Evol Biol. 2012 Aug 30;12:163. doi: 10.1186/1471-2148-12-163 (PMC3502269; doi:10.1186/1471-2148-12-163)
Supplement: Additional file 4 — Figure S1.Alternative phylogenetic trees of insect visual opsins reconstructed by the Maximum likelihood approach. [file 1471-2148-12-163-S4.pdf]

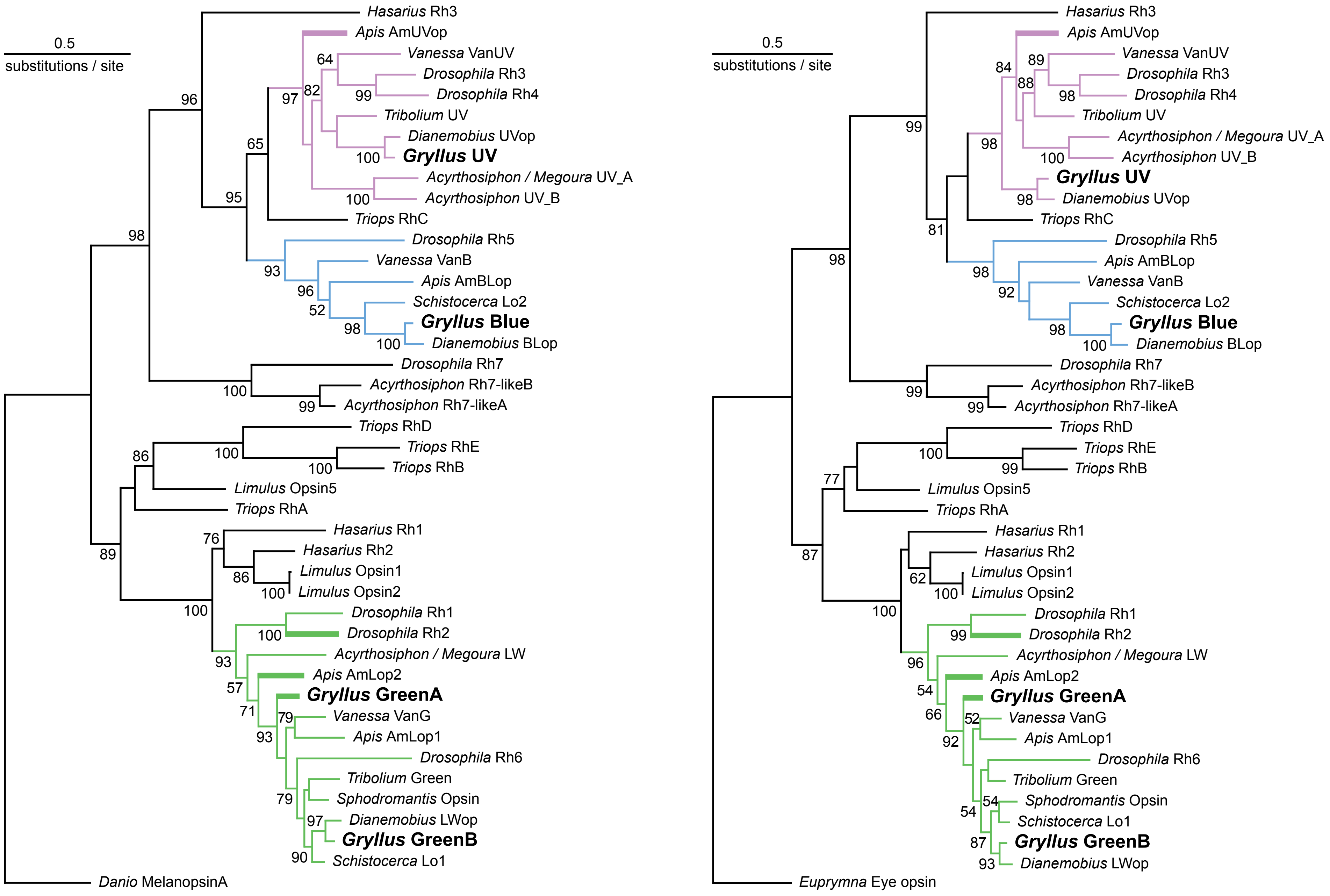

Figure S1 - Alternative phylognetic trees of insect visual opsins reconstructed by the Maximum likelihood approach. As in Fig. 2 but with zebrafish (*Danio rerio*) melanopsin and Hawaiian bobtail squid (*Euprymna scolopes*) eye opsin as outgroups.
